# Primitive Duplicate Hox Clusters in the European Eel's Genome

**DOI:** 10.1371/journal.pone.0032231

**Published:** 2012-02-24

**Authors:** Christiaan V. Henkel, Erik Burgerhout, Daniëlle L. de Wijze, Ron P. Dirks, Yuki Minegishi, Hans J. Jansen, Herman P. Spaink, Sylvie Dufour, Finn-Arne Weltzien, Katsumi Tsukamoto, Guido E. E. J. M. van den Thillart

**Affiliations:** 1 ZF-screens B.V., Leiden, The Netherlands; 2 Institute of Biology, Leiden University, Leiden, The Netherlands; 3 UMR BOREA, CNRS 7208, Muséum National d'Histoire Naturelle, Paris, France; 4 Norwegian School of Veterinary Science, Oslo, Norway; 5 Atmosphere and Ocean Research Institute, The University of Tokyo, Kashiwa, Chiba, Tokyo, Japan; Ecole Normale Supérieure de Lyon, France

## Abstract

The enigmatic life cycle and elongated body of the European eel (*Anguilla anguilla* L., 1758) have long motivated scientific enquiry. Recently, eel research has gained in urgency, as the population has dwindled to the point of critical endangerment. We have assembled a draft genome in order to facilitate advances in all provinces of eel biology. Here, we use the genome to investigate the eel's complement of the Hox developmental transcription factors. We show that unlike any other teleost fish, the eel retains fully populated, duplicate Hox clusters, which originated at the teleost-specific genome duplication. Using mRNA-sequencing and *in situ* hybridizations, we demonstrate that all copies are expressed in early embryos. Theories of vertebrate evolution predict that the retention of functional, duplicate Hox genes can give rise to additional developmental complexity, which is not immediately apparent in the adult. However, the key morphological innovation elsewhere in the eel's life history coincides with the evolutionary origin of its Hox repertoire.

## Introduction

**Figure 1:**
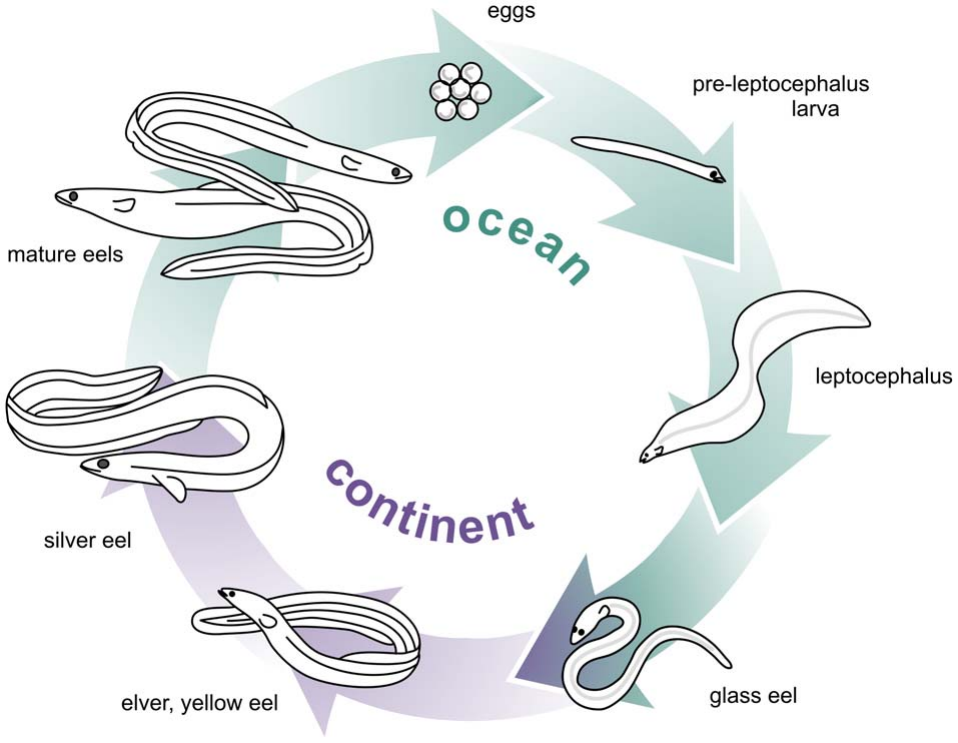
The life cycle of the European eel. After hatching, presumably in the Sargasso Sea, cylindrical larvae develop into leaf-shaped leptocephalus larvae, which after drifting on the Gulf Stream for approximately one year metamorphose into glass eels close to the European coast. The glass eels may stay at the coast or migrate upriver, where they stay as juveniles (elvers and yellow eel) for many years (depending on the region: males 4–6 years, females 8–12 years). Finally, they develop into migrating silver eels; the cause and timing of silvering is not well understood. They mature during or after migration to the spawning grounds.

The life history of the European eel (*Anguilla anguilla* L., 1758) involves two distinct ocean-dwelling larval stages, a protracted juvenile phase in European continental freshwater, and finally sexual maturation coincident with migration to spawning grounds in the Atlantic Ocean, presumably the Sargasso Sea (Figure 1) [1]. The complexity and geographical range of this life cycle have long inspired evolutionary and physiological studies, especially on the structure of the eel's single, randomly mating (panmictic) population [2], interspecific hybridization with the American eel (*A. rostrata*, which shares the same oceanic spawning grounds [3]), its hidden migrations [4]–[6], and the development of fertility [6].

Its catadromous migratory behaviour, long life, serious habitat reduction, pollution, and overfishing may be amongst the causes of the catastrophic collapse of the European eel population observed over the past decades [7]. So far, *Anguilla* species have resisted efforts directed at efficient and sustainable artificial breeding [8]. As knowledge on the eel's genetic makeup is sparse, physiological studies aimed at understanding maturation, reproduction and the sustenance of successive larval stages have not been able to take full advantage of gene expression profiling. In order to alleviate this shortcoming, we have sequenced and assembled its genome.

While the draft genome will be an important tool in reproduction research, it also offers new perspectives for fundamental studies in eel biology, as well as a resource for the comparative interpretation of model fish genomes (e.g. zebrafish and medaka). Here, we investigate its repertoire of Hox genes in a comparative genomics context.

The Hox genes encode transcription factors, which throughout the animal kingdom are involved in the developmental patterning of the body plan. In vertebrates, Hox genes are tightly organized into clusters which exhibit colinearity between gene position and temporal and spatial expression along the primary body axis: genes at the 3′ ends of clusters are expressed earlier in development, and more anterior, than genes at the 5′ ends of clusters [9]. The organization of Hox clusters has been extensively documented for many groups of vertebrates [10].

*A. anguilla* is a member of the superorder Elopomorpha [11], [12], a major teleost group of 856 species [13]. As such, elopomorphs presumably share the inferred whole-genome duplication at the base of the teleost lineage [14], [15]. This teleost-specific genome duplication (TSGD) event is most apparent when considering the Hox genes in extant species [10], [16], [17]. In tetrapods and coelacanths, approximately forty genes are organized in four ancestral vertebrate clusters. In theory, teleosts could have retained eight duplicate clusters. However, whereas tetrapod Hox loci are relatively stable, teleost genomes show dramatic gene loss, such that all species examined in detail retain at most seven of these clusters, each with on average about half their original gene content [9], [10]. A PCR-based survey of the Hox clusters of the Japanese eel *A. japonica* found evidence for the conservation of eight clusters and 34 genes [18].

As the Elopomorpha represent an early branch on the teleost tree [12], the eel Hox gene complement may expose constraints on the evolution of morphological complexity in teleost fish, and in vertebrates in general. Furthermore, analysis of the eel's Hox clusters may shed light on the developmental mechanisms and evolutionary history of its life cycle and body plan. In particular, they may provide evidence regarding the evolutionary novelty of the eel's indirect development.

## Results

### Genome assembly of the European eel

We have sequenced and assembled the genome of a female juvenile *A. anguilla* specimen caught in the brackish Lake Veere, the Netherlands in December 2009. Its haploid genome size was determined to be 1.1 Gbp. Because of the impossibility of breeding *A. anguilla*, no genetic linkage information is available. We therefore employed Illumina Genome Analyzer sequencing technology only in the assembly of a draft genome. Based on a *de novo* genome assembly, we constructed 923 Mbp of scaffolds with a length-weighed median fragment length (N50) of 78 Kbp (Figure S1 and Table S1). An additional 179 Mbp of initial contigs, which are either very small or highly repetitive, were excluded from scaffolding, but included in all further analyses.

### Identification of *Hox* transcripts and genes

**Figure 2:**
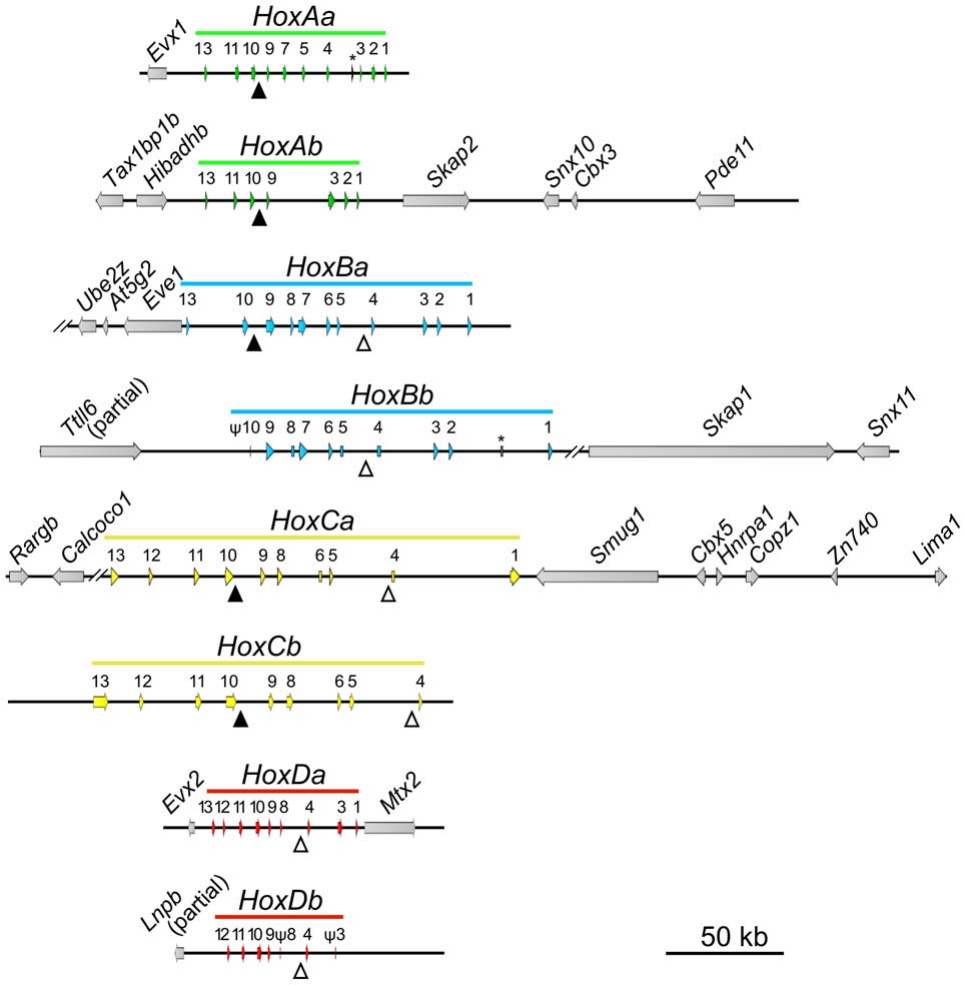
Genomic organization of the Hox gene clusters of the European eel. Scaffolds are indicated by black lines and asterisks represent two gaps between scaffolds. Hox genes are indicated by colored arrows that are numbered according to their paralogous groups. Three pseudogenes are indicated by the symbol ψ. Neighboring genes are indicated by grey arrows. Conserved microRNA genes are indicated by triangles: miR-196 (closed triangles) and miR-10 (open triangles).

To identify *A. anguilla* Hox genes, we used a *de novo* assembled transcriptome of a 27 hours post-fertilization (hpf) embryo of the short-finned eel, *A. australis*. This species is closely related to *A. anguilla* [19], yet produces viable embryos more easily [20]. We compared Hox-like sequences from the transcriptome to the genome assembly using Blast [21], which yielded ten genomic scaffolds (Table S2) that were further examined for the presence of additional genes. This resulted in the identification of 73 Hox genes (twice as many as found in *A. japonica* in a previous study using PCR fragments [18]), including three pseudogenes, organized in eight clusters (Figure 2 and Table S3). The flanking regions of these eight clusters contain an additional 24 predicted genes (Figure 2). No further protein-coding genes were found within the Hox clusters.

Conserved microRNAs were discovered using Blast searches with human and zebrafish homologues (Figure 2). miR-10 is present posterior of Hox4 in six clusters (all except Aa and Ab). miR-196 is found between Hox9 and Hox10 in five clusters (all except Bb, Da and Db). This arrangement is consistent with that found in other vertebrates [22], [23].

### Hox cluster identity

We based preliminary identification of clusters on homology between *A. anguilla* and *Danio rerio* protein sequences and comparisons with all sequences in the NCBI non-redundant protein database (Table S3). Whereas the two *A. anguilla* HoxA clusters can easily be matched to their corresponding HoxAa and HoxAb orthologues in *D. rerio*, each of the two HoxB and HoxC clusters of *A. anguilla* most closely resembles *D. rerio* HoxBa and HoxCa, respectively. Both *A. anguilla* HoxD clusters predictably match *D. rerio* HoxDa only, since the zebrafish HoxDb cluster has lost all protein-coding genes [24].

**Figure 3:**
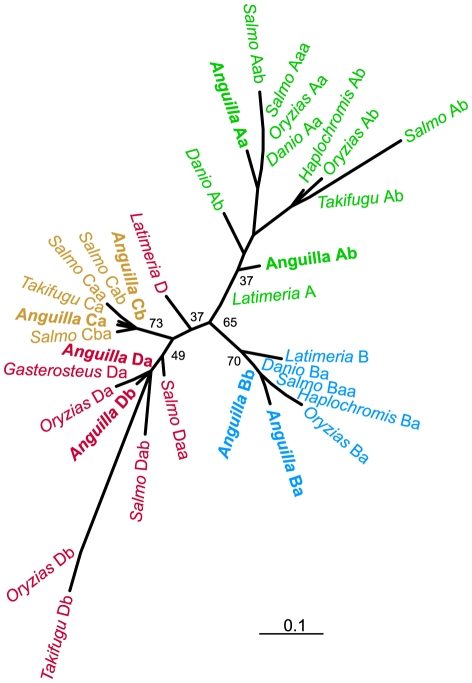
Classification of the European eel Hox clusters. An unrooted phylogenetic tree showing the relationships between *A. anguilla* and fish Hox9 paralogues. Numbers indicate bootstrap support.

**Figure 4:**
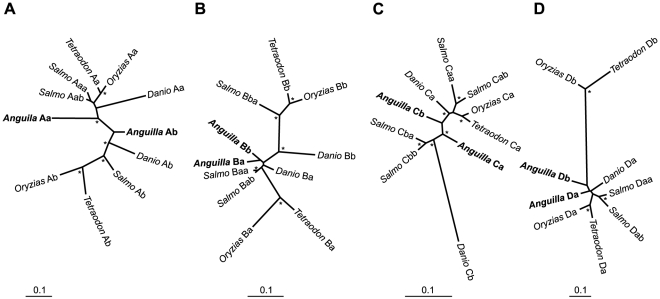
Phylogeny of Hox clusters of the European eel. Unrooted phylogenetic trees based on alignments combining multiple Hox genes per cluster. A) Cluster A relationships, based on HoxA9, HoxA11 and HoxA13 genes. B) Cluster B relationships, based on HoxB1, HoxB5 and HoxB6 genes. C) Cluster C relationships, based on HoxC6, HoxC11, HoxC12 and HoxC13 genes. D) Cluster D relationships, based on HoxD4 and HoxD9 genes. Species included: *A. anguilla*, *Salmo salar* (Atlantic salmon), *Danio rerio* (zebrafish), *Oryzias latipes* (medaka), and *Tetraodon nigroviridis* (green spotted puffer). Asterisks indicate bootstrap support >90%.

To more precisely assign the Hox genes to proper cluster orthologues, we generated unrooted maximum likelihood phylogenetic trees for paralogous group 9 (Figures 3 and S2), of which *A. anguilla* possesses all eight copies. These confirmed the preliminary classification in A, B, C and D paralogous groups, but failed to validate the identity of teleost a and b cluster duplicates (with the exception of HoxAa and HoxAb). Likewise, phylogenetic trees based on multi-gene alignments do not conclusively indicate either a or b cluster membership for HoxB, HoxC and HoxD (Figure 4). In general, there appears to be a lack of sequence divergence between eel Hox gene duplicates, which makes classification based on coding sequence alone inaccurate.

**Figure 5:**
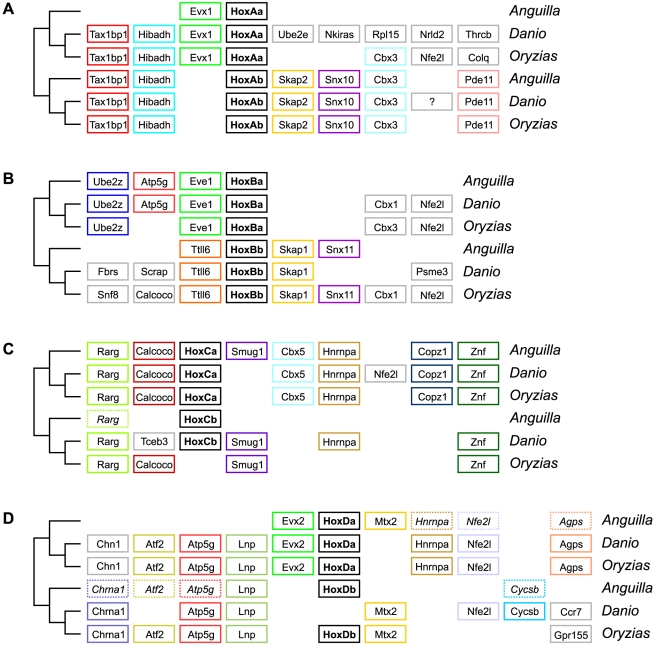
Synteny around Hox clusters. Conservation of flanking genes supports the classification of *A. anguilla* clusters into different orthologous subgroups. The eel clusters and up to seven flanking genes are compared with the genomic organization in zebrafish (*Danio rerio*) and medaka (*Oryzias latipes*). Coloured box outlines indicate preserved synteny between eel and the two other species, dotted outlines denote flanking genes found on extended eel scaffolds (see Methods). Interpretation should take into account residual synteny between a and b paralogous clusters. Limited data is available on HoxCb (lost in *O. latipes*, possibly misassembled in *D. rerio*) and HoxDb (lost in *D. rerio*) clusters.

Final orthologous relationships could only be established on the basis of conserved local synteny between Hox clusters and flanking genes (Figure 5). In addition to both HoxA clusters, eel HoxBa and HoxBb appear orthologous with their respective teleost equivalents. This identification is further supported by the absence of miR-196 from both *D. rerio* and *A. anguilla* HoxBb clusters. The affinities of HoxC and HoxD duplicates remain difficult to resolve because of conserved synteny around a and b cluster duplicates, and extensive cluster reduction and deletion in other teleosts (Figure 5c, d).

### Hox gene expression

**Figure 6:**
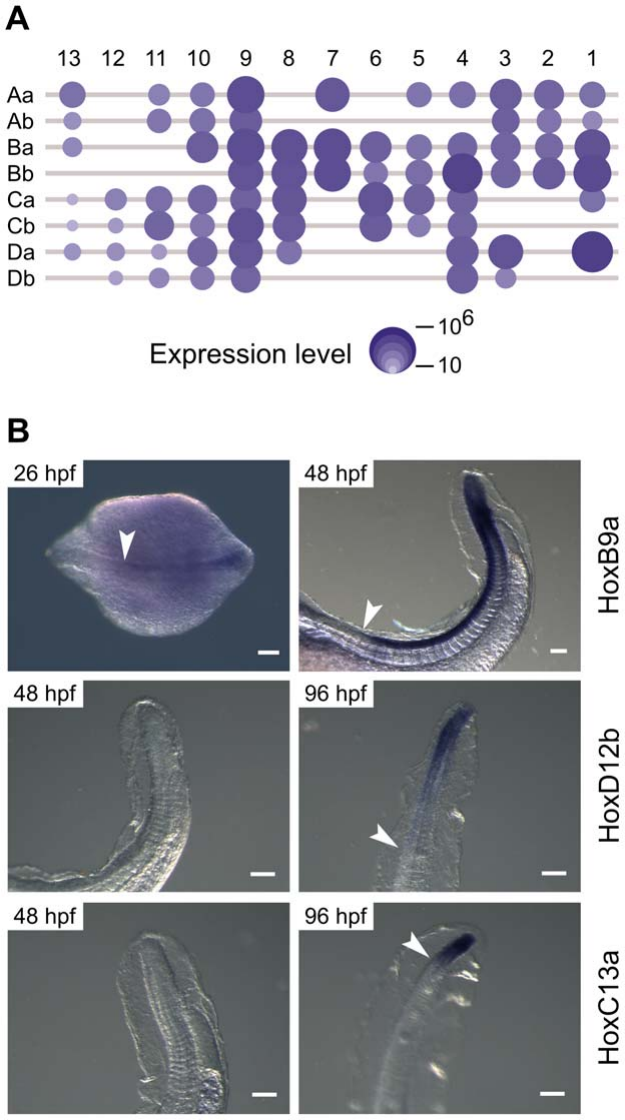
Hox gene expression in *A. australis* embryos. A) mRNA-seq-based gene expression in a 27 hpf embryo. B) Whole mount *in situ* hybridizations showing the expression of *HoxB9a*, *HoxD12b* and *HoxC13a*. *HoxB9a* expression can be detected in 26 hpf (dorsal view) and 48 hpf (lateral tail region view) embryos. *HoxD12b* and *HoxC13a* display expression in the tail region (lateral views) at 96 hpf, but not at 48 hpf. White arrowheads indicate anterior expression boundaries. Scale bars correspond to 100 µm.

In order to confirm the transcriptional activity of the Hox genes, we determined relative expression levels by aligning transcriptomic reads of the 27 hpf embryo against the Hox protein-coding regions (Figure 6a). Transcriptome reads mapped unambiguously to 71 out of 73 Hox genes, including one pseudogene (*ψHoxD3b*), suggesting that all *A. anguilla* Hox protein-coding genes are functional. The relative expression levels vary over five orders of magnitude with the lowest expression observed for the posterior paralogous groups 12 and 13, and the highest expression for paralogous groups 7–9, but with particularly high expression levels for *HoxB1a*, *HoxB1b*, *HoxB4b* and *HoxD1a*.

Full mRNA-seq read alignment to the entire Hox clusters indicated that transcriptional activity is not restricted to protein coding regions (Figure S3). In fact, intergenic expression sometimes exceeds intragenic levels, supporting the observation that complete Hox clusters function as meta-genes [9], [25].

At 27 hpf, expression of posterior Hox genes is very low (Figure 6a). We therefore confirmed transcriptional activity of posterior Hox paralogues by whole mount *in situ* hybridizations (Figure 4b). *HoxB9a* is expressed at 26 and 48 hpf, with an anterior expression boundary coinciding with somite number 5/6. Expression of *HoxD12b* and *HoxC13a* is not yet detectable at 48 hpf, but clearly visible at 96 hpf with anterior expression boundaries located at somite numbers 65/70 and 90/95 for *HoxD12b* and *HoxC13a*, respectively. For these Hox genes, expression in the eel embryo appears to conform to the expected spatio-temporal pattern (colinearity between cluster organization and developmental timing and localization), with expression of Hox12 and Hox13 paralogues appearing later in development, and more posterior than Hox9.

### The evolution of Hox cluster organization

**Figure 7:**
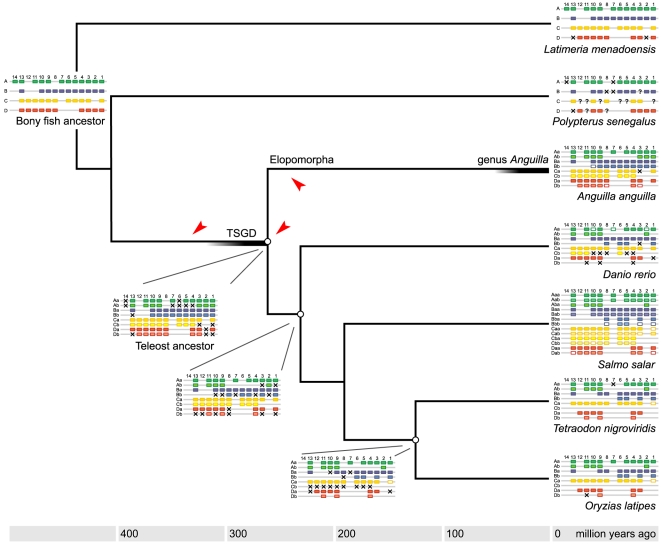
Model for the evolution of teleost Hox gene organization. Schematic Hox clusters [10], [26], [52] are superimposed on a species phylogeny with estimates of divergence times [53], [54] – which vary considerably between studies [33]. Ancestral architectures are inferred on the basis of maximum parsimony, i.e. the number of cluster duplications and gene loss events is minimized. *Salmo salar* (Atlantic salmon) has presumably lost several duplicate clusters [26] (not shown). Deduced gene loss in a lineage is illustrated by a cross, question marks denote uncertainty about cluster gene content in the pre-TSGD actinopterygian *Polypterus senegalus* (bichir). Arrows indicate the possible origins of the leptocephalus body plan.

The early branching of the Elopomorpha from the main teleost trunk allows a new reconstruction of ancestral Hox cluster architectures (Figure 7), which are strongly constrained by the limited organizational divergence between eel HoxB, C and D duplicates.

Since teleost fish are believed to have experienced the TSGD event early in their evolutionary history [14], [15], their genomes should in theory possess up to eight cluster duplicates. However, all teleosts examined in detail retain at most seven clusters of protein-coding genes [9]: a HoxC duplicate was lost in the lineage leading to medaka and pufferfish, a HoxD duplicate in the lineage represented by zebrafish. The high number of clusters in salmon is the result of relatively recent further duplications [26].

The main teleost lineages diverged briefly after the TSGD [16]. The reconstruction in Figure 7 demonstrates that nearly all post-duplication gene loss events in the eel's ancestry occurred within this interval, followed by millions of years of stasis. Only the HoxAb cluster appears to have accumulated major changes in pre-branching, post-genome duplication teleosts. Alternative hypotheses, in which a whole-genome duplication is not shared between elopomorphs and advanced teleosts, or in which the genome duplication is followed by successive deletion and duplication of specific clusters in the eel, are less parsimonious and not consistent with local conservation of synteny (Figure 5).

## Discussion

Two rounds of Hox cluster duplications in chordates are believed to be responsible for important vertebrate novelties (e.g. brains, heads, jaws) and increases in complexity [27]. A plausible mechanism invokes a temporary relaxation of meta-genic cluster constraints after duplication, paving the way for innovation [28], [29]. In contrast, the TSGD-associated third duplication of vertebrate Hox clusters theoretically endowed teleost fish not with additional complexity within individuals, but with increased prospects for morphological diversification between individuals and species [9], [10]. In support of this hypothesis, advanced teleosts have extensively pruned their Hox surplus, leading to significant diversity in cluster structure (Figure 7). In all examined representatives (with the exception of salmon [26]), the residual number of Hox genes is not much higher than the non-duplicated count in tetrapods. The resulting teleost Hox cluster architectures have been interpreted as an evolutionary choice for developmental flexibility in a trade-off with robustness [9]. By proving that it is possible for a vertebrate to stably preserve eight densely populated (Figure 2) and functional (Figure 6) Hox clusters, the eel genome presents an exception to these models, and a third alternative in the evolution of vertebrate complexity.

For hundreds of millions of years, *A. anguilla* and its ancestors have maintained the highest ontogenic potential of any vertebrate, indicative of continuous selective pressure. However, as adults, they do not display markedly more complex bodies than other fish or tetrapods. The eel's distinctive life cycle and body plan suggest three (not mutually exclusive) explanations for this cryptic complexity.

Hox genes are involved in the primary patterning of the body axis, which implies a functional role for *A. anguilla*'s Hox surplus in axial elongation. Alterations in Hox genes have been associated with elongated body plans [30], [31], however the changes observed are of a regulatory nature, and do not involve extra genes. For example, elongation of the body axis in snakes has been linked to a spatial relaxation in the posterior end of Hox clusters facilitated by the insertion of transposable elements between genes [31]. In addition, even the elongate members of the Elopomorpha (which also includes non-elongated tarpons, bonefish and others) display considerable diversity in the developmental mechanisms resulting in axial lengthening [32]. Hence, the eel's adult body plan cannot explain the preservation of primitive Hox clusters between the TSGD (226–316 million years ago [33]) and the origin of the genus *Anguilla*, estimated at 20–50 million years ago [19]. Similarly, if the European eel may at present experience singular evolutionary forces because of its panmictic population [2], any explanation these offer does not extend beyond the genus *Anguilla* of freshwater eels [34].

Even if for most of their lives eels are eel-shaped, as ocean-dwelling larvae [35] their body plan is radically different (Figure 1). In fact, until the late nineteenth century, these large, long-lived, laterally compressed leptocephali were considered to be autonomous pelagic species [36]. Fully transparent and slowly metabolizing, a leptocephalus provides considerable survival benefits [37], [38]. After approximately one year, they undergo a dramatic metamorphosis [39], including extensive tissue remodelling and shortening of the body, resulting in cylindrical juveniles. In the early embryos investigated here (Figure 6), nearly all Hox genes are expressed and presumably functionally involved in determining cell fate. Logically, a high gene and cluster count can be explained by the assumption that the eel's two body plans are simultaneously outlined at this stage.

Leptocephali are the fundamental developmental innovation shared by all Elopomorpha [11]–[13], and therefore arose either before or soon after the TSGD, or at the base of the lineage (arrows in Figure 7). The last alternative is the most parsimonious (no loss of developmental complexity in advanced teleosts), especially since no member of the Elopomorpha is known to have ever discarded the leptocephalous larval stage [11], [13]. Regardless, either of the post-TSGD origins is compatible with an intercalation model of indirect development [40], in which a temporary excess of developmental potential was permanently recruited for the conception of an additional body plan. Although speculative, an explanation invoking the morphological challenges associated with a complex life history is consistent with the stable high Hox gene and cluster count found in the anadromous Atlantic salmon [26].

Further functional studies on eel development will become possible once *A. anguilla*'s life cycle can be completed in captivity. In particular, there exists considerable variation in development (timing, number of somites) between leptocephali of related and interbreeding *Anguilla* species [1], [35], which can only be studied when these larvae can be raised under controlled conditions [8], [41].

## Methods

### Eel embryos

Wild female and male silver short-finned silver eels (*A. australis*) from Lake Ellesmere, New Zealand, were held together in a 2,300 L recirculation system with seawater (30 ppt salinity) at 21°C. Sexual maturation was induced as described [20]. Briefly, males received nine weekly injections with 250 IU human chorionic gonadotropin and females were injected once a week with 20 mg salmon pituitary extract. Eggs and milt were stripped and the eggs were dry fertilized. Embryos were reared in glass beakers with UV-sterilized seawater (35 ppt) at 21°C. At 26, 48 and 96 hpf embryos were fixed in 4% paraformaldehyde and stored in 100% methanol.

Total RNA was isolated from 27 hpf embryos using the Qiagen miRNeasy kit according to the manufacturer's instructions (Qiagen GmbH, Hilden, Germany), and analyzed with an Agilent Bioanalyzer 2100 total RNA Nano series II chip (Agilent, Santa Clara). A transcriptome library was prepared from 10 µg total RNA, using the Illumina mRNA-Seq Sample Preparation Kit according to the manufacturer's instructions (Illumina Inc., San Diego, USA).

### Genome size determination

Blood samples taken from two eels (*A. anguilla* and *A. australis*) were washed with physiological salt and fixed in cold ethanol. Prior to analysis the cells were collected, resuspended in physiological salt and stained with propidium iodide. After 30 minutes of incubation the cells were analyzed by FACS, using human blood cells as a size reference (3.05 Gbp haploid). The eel genome size was calculated by (human size)/(mean fluorescence human)×(mean fluorescence eel). Both *Anguilla* genomes were determined to be 1.1 Gbp in size (haploid).

### Genomic DNA libraries

Genomic DNA was isolated from blood of a female yellow European eel (*A. anguilla*, caught in Lake Veere, The Netherlands) using the Qiagen Blood and Tissue DNeasy kit according to the manufacturer's description. Paired-end libraries were prepared from 5 µg of isolated gDNA using the Paired-End Sequencing Sample Prep kit according to the manufacturer's description. Either a 200 bp band or a 600 bp band was cut from the gel (libraries PE200 and PE600, Table S1). After amplification for 10 cycles the resulting libraries were analyzed with a Bioanalyzer 2100 DNA 1000 series II chip.

Mate pair libraries were prepared from 10 µg of isolated gDNA using the Mate Pair Library Prep Kit v2 (Illumina Inc.). Either a 3,000 bp band or a 10,000 bp band was cut from gel (libraries MP3K and MP10K, Table S1). After the first gel purification the fragment length was analyzed using a Agilent Bioanalyzer 2100 DNA 12000 chip. After circularization, shearing, isolation of biotinylated fragments, and amplification, the 400 to 600 bp fraction of the resulting fragments was isolated from gel. Finally, the libraries were examined with an Agilent Bioanalyzer 2100 DNA 1000 series II chip.

### Illumina sequencing

All libraries were sequenced using an Illumina GAIIx instrument according to the manufacturer's description. Genomic paired-end libraries were sequenced with a read length of 2×76 nucleotides (to ∼20-fold genome coverage), genomic mate-pair libraries with a read length of 2×51 nucleotides (to ∼33-fold genome span), and the mRNA-Seq library with a read length of 2×76 nucleotides (Table S1). Image analysis and base calling was done by the Illumina pipeline.

### Genome assembly

Sequencing reads from both paired-end libraries were used in building the initial contigs (Figure S1). Both sets were preprocessed to eliminate low quality and adapter contamination. Whenever possible, PE200 pairs were merged into longer single reads. For initial contig assembly, we employed the De Bruijn graph-based *de novo* assembler implemented in the CLC bio Genomics Workbench version 3.6.5 (CLC bio, Aarhus, Denmark). A run with a k-mer length of 25 nt resulted in an assembly a total length of 969 Mbp and a contig N50 of 1672 bp.

Initial contigs were oriented in larger supercontigs (scaffolds) using SSPACE [42]. In scaffolding the contigs, we decided to exclude low-quality and highly repetitive contigs as much as possible. SSPACE was used in a hierarchical fashion, employing first links obtained from the PE600 library to generate intermediate supercontigs, which were used as input for subsequent runs with links from the MP3K and MP10K libraries, respectively. At each stage, a minimum of three non-redundant links was required to join two contigs. This procedure resulted in a final scaffold set with a total length of 923 Mbp and an N50 of 77.8 Kbp (Table S1). AUGUSTUS (version 2.4) was used to predict genes [43], which were provisionally annotated using Blast2GO (version 2.4.8) [44]. The draft assembly is available at www.eelgenome.org.

In order to obtain more information on flanking genes for the analysis of conserved synteny (Figure 5), scaffolds were subjected to a further round of linking by SSPACE using reduced stringency (two instead of three non-redundant links required to join scaffolds). This resulted in extended scaffolds with an N50 of 169 Kbp.

### Hox genes

Hox contigs in the short-finned eel embryonic transcriptome (generated using CLC bio's *de novo* assembler) were identified via Blast [21] searches at the NCBI website (www.ncbi.nlm.nih.gov). European eel genomic scaffolds were annotated using CLC bio's DNA Workbench. Remaining Hox genes and genes flanking the Hox clusters were identified using Blast, based on AUGUSTUS/Blast2GO predictions. Annotated Hox scaffolds have been submitted to GenBank (accession numbers JF891391–JF891400).

MicroRNAs were identified by Blast using *H. sapiens* and *D. rerio* miR-10 and miR-196 sequences (precursors and mature) retrieved from miRBase release 18 (www.mirbase.org, [45]).

### Phylogenetic methods

Species and Hox gene accession numbers used are listed in Table S4. Amino acid sequences of Hox genes were aligned using Clustal X [46] and checked manually. After excluding ambiguous alignments, ProtTest 2.4 [47] was used to choose an optimum substitution model, based on the Akaike information criterion. The aligned sequences were subjected to maximum likelihood analysis using RAxML version 7.2.6 [48] with 1000 rapid bootstrap replicates (-f a option).

For the analysis of Hox9 genes (Figure 3), 70 aligned residues were used and analyzed using a JTT+I+Γ model [49]. All other alignments were fitted using a JTT+Γ model. The multi-gene analyses of HoxA, HoxB, HoxC and HoxD (Figure 4) were based on alignments of 427, 493, 935 and 308 amino acid residues, respectively. The phylogenetic trees of sarcopterygian and actinopterygian Hox9 paralogues (Figure S2) were based on 151 (HoxA9), 210 (HoxB9), 248 (HoxC9), and 136 (HoxD9) residues.

Synteny was analyzed using *D. rerio* and *O. latipes* genomic contexts extracted from Ensembl release 65 (www.ensembl.org), based on the Zv9 and MEDAKA1 genome assemblies, respectively (Table S5). Pairwise alignments were generated by NCBI tblastx and analyzed using genoPlotR [50].

### Whole mount *in situ* hybridization

Chromosomal DNA was isolated from *A. australis* blood using a DNeasy Blood & Tissue Kit (Qiagen). Riboprobe template fragments, including a T7 RNA polymerase promoter, were PCR amplified from chromosomal DNA using the following primer sets: *HoxB9a* forward (5′-TGAAACCGAAGACCCGAC-3′), *HoxB9a* reverse (5′-GAAATTAATACGACTCACTATAGGGCTGAGGAAGACTCCAA), *HoxD12b* forward (5′-TAATCTTCTCAGTCCTGGCTATG-3′), *HoxD12b* reverse (5′-GAAATTAATACGACTCACTATAGATCCAAGTTTGAAAATTCATATTTGC-3′), *HoxC13a* forward (5′-CACCTTGATGTACGTGTATGAAAA-3′), *HoxC13a* reverse (5′-GAAATTAATACGACTCACTATAGGCTCCGTGTATTTCTCTGACG-3′). Digoxygenin-labelled riboprobes were made according to standard protocols using T7 RNA polymerase. Whole mount *in situ* hybridization with labelled riboprobes was performed at 70°C, according to a slightly modified version of a standard protocol [51]. Hybridizing riboprobes were made visible using anti-Digoxigenin AP and BM Purple AP substrate (Roche). Stained embryos were bleached using hydrogen peroxide (Sigma-Aldrich) and photographed using a Leica M205 FA stereo microscope.

## Supporting Information

Figure S1 **Genome assembly pipeline.** See Methods section for details.
(TIF)

Figure S2 **Unrooted maximum likelihood phylogenetic trees of actinopterygian and sarcopterygian Hox9 genes.** See Methods section for details. Sequences used are listed in Table S4.
(TIF)

Figure S3 **Meta-genic expression of Hox clusters.** mRNA-seq reads of the *A. australis* embryo were aligned to entire Hox-containing scaffolds, demonstrating large amounts of mRNA production from intronic and intergenic regions.
(TIF)

Table S1 **Statistics of European eel genome and short-finned embryonic eel transcriptome.**
(DOC)

Table S2 **Hox transcriptome contigs.** All *de novo* assembled Hox contigs of a 27-hour *A. australis* embryo map to ten *A. anguilla* genome scaffolds.
(DOC)

Table S3 ***A. anguilla*** **Hox genes.** Complete list of *A. anguilla* Hox genes, predicted protein sizes, matching *A. australis* embryo contigs and best blastp hits.
(DOC)

Table S4 **Hox genes used in phylogeny reconstruction.** List of the Hox gene sequences used in this study.
(DOC)

Table S5 **Hox clusters used in synteny analysis.** Genomic locations of *D. rerio* and *O. latipes* Hox clusters. HoxCb is absent from *O. latipes*, and HoxDb from *D. rerio*. However, the genomic loci can still be identified based on the presence of flanking gene duplicates or conserved microRNA (*D. rerio* HoxDb).
(DOC)
