# Supplementary figures and images for: Primitive Duplicate Hox Clusters in the European Eel's Genome
Source: PLoS One. 2012 Feb 24;7(2):e32231. doi: 10.1371/journal.pone.0032231 (PMC3286462; doi:10.1371/journal.pone.0032231)

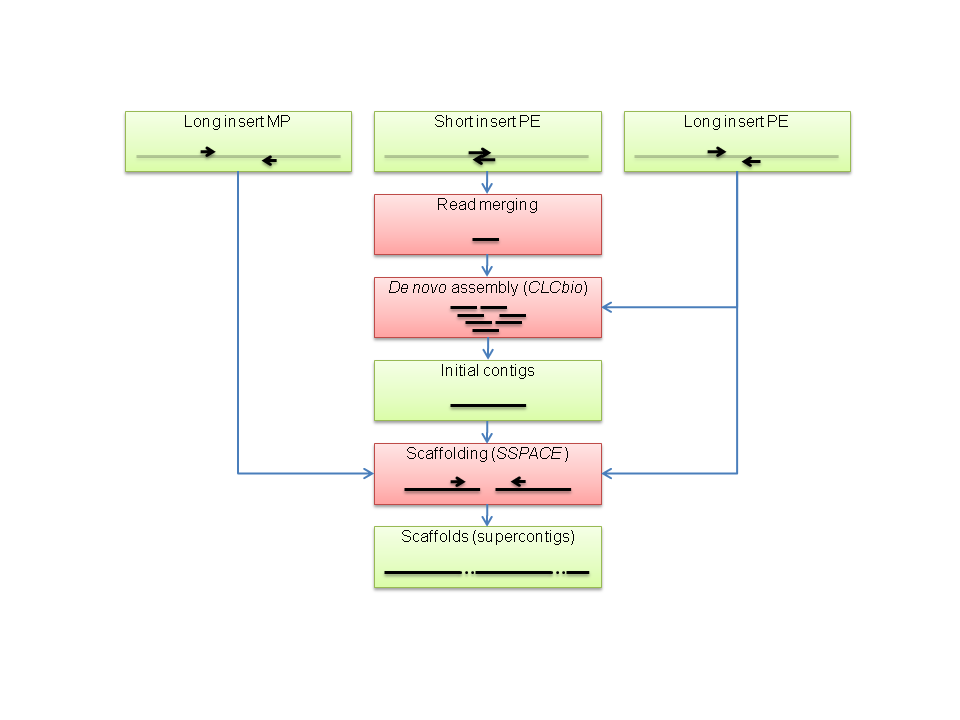

Supplement: Figure S1 — Genome assembly pipeline. See Methods section for details. (TIF) [file pone.0032231.s001.tif]

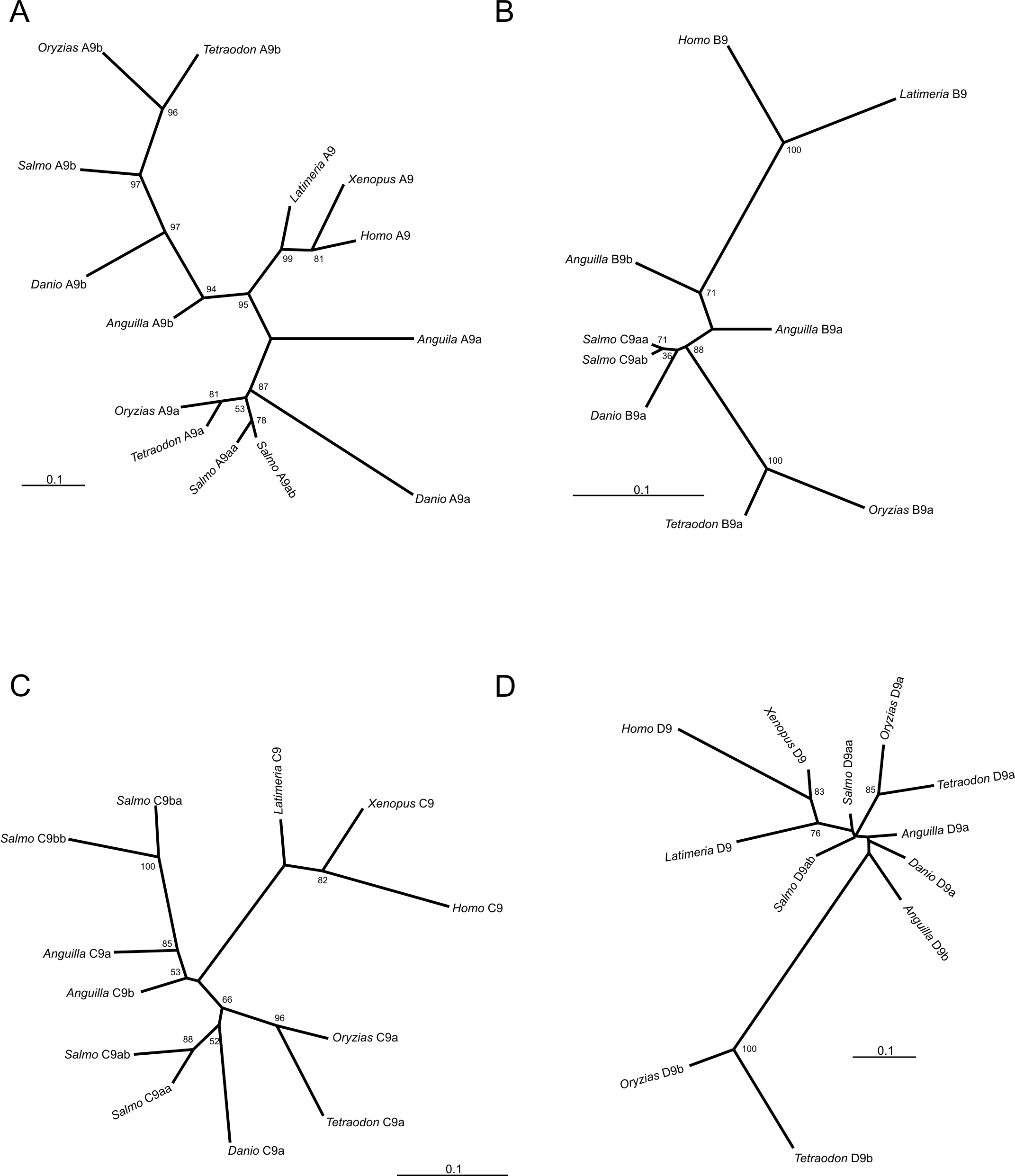

Supplement: Figure S2 — Unrooted maximum likelihood phylogenetic trees of actinopterygian and sarcopterygian Hox9 genes. See Methods section for details. Sequences used are listed in Table S4. (TIF) [file pone.0032231.s002.tif]

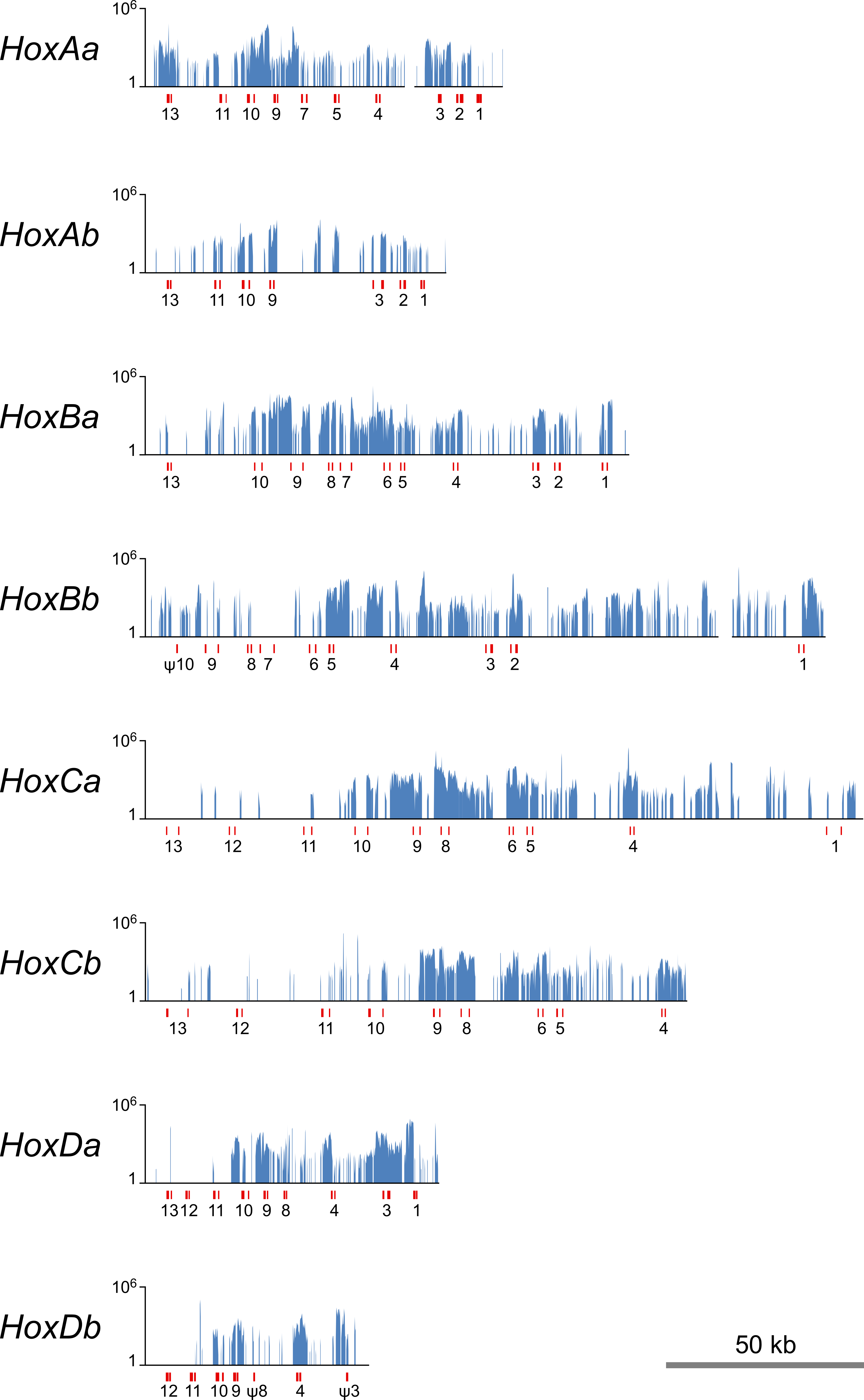

Supplement: Figure S3 — Meta-genic expression of Hox clusters. mRNA-seq reads of the A. australis embryo were aligned to entire Hox-containing scaffolds, demonstrating large amounts of mRNA production from intronic and intergenic regions. (TIF) [file pone.0032231.s003.tif]
