# Supplementary material for: Primitive Duplicate Hox Clusters in the European Eel's Genome
Source: PLoS One. 2012 Feb 24;7(2):e32231. doi: 10.1371/journal.pone.0032231 (PMC3286462; doi:10.1371/journal.pone.0032231)
Supplement: Table S1 — Statistics of European eel genome and short-finned embryonic eel transcriptome. (DOC) [file pone.0032231.s004.doc]

**Table S1.** Statistics of European eel genome and short-finned embryonic eel transcriptome.

**Genome libraries summary**

| **Sequencing library** | **Fragment size** | **Read length** | **Lanes** | **Raw data** | **Coverage*** | **Span*** |
| --- | --- | --- | --- | --- | --- | --- |
| PE200 | <300 bp | 2×76 nt | 3 | 12.4 Gbp | 11.3× | n.a. |
| PE600 | 150–950 bp | 2×76 nt | 2 | 8.9 Gbp | 8.1× | 2.4× |
| MP3K | 3 Kbp ± 50% | 2×51 nt | 3* | 10.4 Gbp | n.a. | 22.7× |
| MP10K | 8 Kbp ± 25 % | 2×51 nt | 1* | 3.7 Gbp | n.a. | 7.4× |

* Coverage: average fold coverage assuming a 1.1 Gbp genome
 Span: average fold coverage of non-redundant entire fragments useful for scaffolding

Assembly stages summary

| **Description** | **Sum** | **Number of sequences** | **Smallest fragment** | **Largest fragment** | **N50*** |
| --- | --- | --- | --- | --- | --- |
| Assembly of PE200 | 927 Mbp | 1.66 M | 100 bp | 25.0 Kbp | 1067 bp |
| Assembly of PE200 + PE600 (final contigs) | 969 Mbp | 1.52 M | 100 bp | 53.8 Kbp | 1672 bp |
| Large, reliable contigs used for scaffolding | 790 Mbp | 602 K | 201 bp | 53.8 Kbp | 2259 bp |
| Small, unreliable and repetitive contigs | 179 Mbp | 918 K | 100 bp | 19.5 Kbp | 192 bp |
| After scaffolding with PE600 | 813 Mbp | 321 K | 201 bp | 161.0 Kbp | 6.9 Kbp |
| After scaffolding with MP3K | 894 Mbp | 195 K | 201 bp | 1.67 Mbp | 48.6 Kbp |
| After scaffolding with MP10K (final scaffolds) | 923 Mbp | 186 K | 201 bp | 2.05 Mbp | 77.6 Kbp |

* N50: length-weighed median fragment length. In other words, 50% of the sequence in a
 set is made up of fragments of at least this size.

Transcriptome library summary

| **Sample** | **Sequencing** | **Read length** | **Number of reads** | **Raw data** |
| --- | --- | --- | --- | --- |
| *A. australis* embryo* | 1 lane Illumina GAIIx | 2×76 nt | 2×33.2 M | 5.0 Gbp |
