# Supplementary material for: Primitive Duplicate Hox Clusters in the European Eel's Genome
Source: PLoS One. 2012 Feb 24;7(2):e32231. doi: 10.1371/journal.pone.0032231 (PMC3286462; doi:10.1371/journal.pone.0032231)
Supplement: Table S2 — Hox transcriptome contigs. All de novo assembled Hox contigs of a 27-hour A. australis embryo map to ten A. anguilla genome scaffolds. (DOC) [file pone.0032231.s005.doc]

**Table S2.** All *de novo* assembled *Hox* contigs of a 27-hour *A. australis* embryo map to ten *A. anguilla* genome scaffolds.

| *A. australis* embryonic transcriptome contig number | Matching *A. anguilla* genome scaffold number (>99% identity) | Best *Danio rerio* blastn hit (E-value) | Best *Danio rerio* blastn hit (Ensembl Gene ID) | Best *Danio rerio* blastn hit (associated gene name) |
| --- | --- | --- | --- | --- |
| 52084 | 84 | 1,04606E-71 | ENSDARG00000056819 | *hoxa9b* |
| 106765 | 84 | 3,6423E-36 | ENSDARG00000031337 | *hoxa10b* |
| 17128 | 84 | 1,88261E-25 | ENSDARG00000023031 | *hoxa2b* |
| 130037 | 84 | 4,09681E-17 | ENSDARG00000007009 | *hoxa11b* |
| 34435 | 84 | 1,04466E-13 | ENSDARG00000007009 | *hoxa11b* |
| 55834 | 84 | 3,49126E-12 | ENSDARG00000059280 | *hoxd3a* |
| 15869 | 84 | 7,55192E-11 | ENSDARG00000059267 | *hoxd11a* |
| 181614 | 290 | 2,23349E-89 | ENSDARG00000070338 | *hoxc4a* |
| 144813 | 290 | 9,84535E-87 | ENSDARG00000070346 | *hoxc8a* |
| 78426 | 290 | 2,10803E-69 | ENSDARG00000070340 | *hoxc5a* |
| 143448 | 290 | 8,81306E-57 | ENSDARG00000070343 | *hoxc6a* |
| 54638 | 290 | 1,06154E-42 | ENSDARG00000070338 | *hoxc4a* |
| 52095 | 290 | 2,70451E-38 | ENSDARG00000070350 | *hoxc10a* |
| 55069 | 290 | 2,20139E-33 | ENSDARG00000070348 | *hoxc9a* |
| 102927 | 290 | 2,89853E-14 | ENSDARG00000070346 | *hoxc8a* |
| 7133 | 290 | 1,06808E-12 | ENSDARG00000056027 | *hoxb8a* |
| 69704 | 290 | 7,26544E-11 | ENSDARG00000070346 | *hoxc8a* |
| 104311 | 330 | 4,7308E-129 | ENSDARG00000010630 | *hoxb6a* |
| 179338 | 330 | 4,56147E-67 | ENSDARG00000056023 | *hoxb9a* |
| 75377 | 330 | 3,38824E-66 | ENSDARG00000013057 | *hoxb5a* |
| 58468 | 330 | 5,53893E-56 | ENSDARG00000029263 | *hoxb3a* |
| 103225 | 330 | 1,51006E-52 | ENSDARG00000054025 | *hoxb8b* |
| 80712 | 330 | 1,99038E-47 | ENSDARG00000013533 | *hoxb4a* |
| 148360 | 330 | 5,00933E-46 | ENSDARG00000029263 | *hoxb3a* |
| 86079 | 330 | 1,7983E-43 | ENSDARG00000054030 | *hoxb5b* |
| 51405 | 330 | 5,01106E-34 | ENSDARG00000054033 | *hoxb1b* |
| 102356 | 330 | 3,50438E-32 | ENSDARG00000013057 | *hoxb5a* |
| 181054 | 330 | 3,94261E-29 | ENSDARG00000029263 | *hoxb3a* |
| 53043 | 330 | 3,42497E-28 | ENSDARG00000013533 | *hoxb4a* |
| 86197 | 330 | 1,08922E-25 | ENSDARG00000013533 | *hoxb4a* |
| 51680 | 330 | 1,15456E-25 | ENSDARG00000011579 | *hoxb10a* |
| 174143 | 330 | 6,01089E-22 | ENSDARG00000013057 | *hoxb5a* |
| 180588 | 330 | 1,61995E-20 | ENSDARG00000023031 | *hoxa2b* |
| 46704 | 330 | 1,782E-20 | ENSDARG00000013057 | *hoxb5a* |
| 77961 | 330 | 4,44882E-19 | ENSDARG00000013057 | *hoxb5a* |
| 145354 | 330 | 2,61987E-16 | ENSDARG00000070343 | *hoxc6a* |
| 16208 | 330 | 5,57724E-16 | ENSDARG00000013057 | *hoxb5a* |
| 50994 | 448 | 5,67028E-28 | ENSDARG00000054033 | *hoxb1b* |
| 53472 | 472 | 8,20869E-98 | ENSDARG00000029263 | *hoxb3a* |
| 50801 | 472 | 1,14867E-52 | ENSDARG00000000175 | *hoxb2a* |
| 146468 | 472 | 4,81704E-45 | ENSDARG00000056027 | *hoxb8a* |
| 100250 | 472 | 1,29316E-42 | ENSDARG00000013057 | *hoxb5a* |
| 63828 | 472 | 1,08229E-41 | ENSDARG00000056023 | *hoxb9a* |
| 146756 | 472 | 7,78491E-28 | ENSDARG00000056027 | *hoxb8a* |
| 70145 | 472 | 1,07356E-24 | ENSDARG00000013533 | *hoxb4a* |
| 56431 | 472 | 4,22574E-22 | ENSDARG00000056030 | *hoxb7a* |
| 81056 | 472 | 1,97775E-20 | ENSDARG00000013057 | *hoxb5a* |
| 63358 | 472 | 2,86668E-20 | ENSDARG00000056030 | *hoxb7a* |
| 66055 | 472 | 3,47592E-17 | ENSDARG00000056023 | *hoxb9a* |
| 32927 | 472 | 3,82434E-13 | ENSDARG00000010630 | *hoxb6a* |
| 148750 | 472 | 2,27892E-10 | ENSDARG00000026513 | *hoxb6b* |
| 104558 | 513 | 0 | ENSDARG00000070338 | *hoxc4a* |
| 143804 | 513 | 2,2099E-104 | ENSDARG00000070346 | *hoxc8a* |
| 103504 | 513 | 2,61378E-59 | ENSDARG00000070343 | *hoxc6a* |
| 93391 | 513 | 6,6982E-56 | ENSDARG00000070350 | *hoxc10a* |
| 144798 | 513 | 1,29026E-39 | ENSDARG00000070348 | *hoxc9a* |
| 108663 | 513 | 2,80822E-38 | ENSDARG00000070340 | *hoxc5a* |
| 62505 | 513 | 4,05108E-35 | ENSDARG00000070338 | *hoxc4a* |
| 179450 | 513 | 5,63105E-25 | ENSDARG00000070348 | *hoxc9a* |
| 104012 | 513 | 6,45752E-24 | ENSDARG00000070346 | *hoxc8a* |
| 188883 | 513 | 6,82966E-23 | ENSDARG00000070340 | *hoxc5a* |
| 45462 | 513 | 9,8259E-10 | ENSDARG00000070340 | *hoxc5a* |
| 180487 | 905 | 8,3061E-176 | ENSDARG00000059280 | *hoxd3a* |
| 56200 | 905 | 3,1078E-161 | ENSDARG00000059276 | *hoxd4a* |
| 55695 | 905 | 5,57612E-67 | ENSDARG00000057859 | *hoxd10a* |
| 51991 | 905 | 2,62469E-41 | ENSDARG00000059274 | *hoxd9a* |
| 125941 | 905 | 1,10091E-23 | ENSDARG00000057859 | *hoxd10a* |
| 84846 | 905 | 1,10915E-15 | ENSDARG00000054025 | *hoxb8b* |
| 33331 | 905 | 1,08008E-12 | ENSDARG00000057859 | *hoxd10a* |
| 57327 | 1005 | 5,04898E-56 | ENSDARG00000059276 | *hoxd4a* |
| 53985 | 1005 | 1,0198E-34 | ENSDARG00000059274 | *hoxd9a* |
| 56644 | 1005 | 5,21932E-33 | ENSDARG00000059276 | *hoxd4a* |
| 189155 | 1005 | 6,71159E-16 | ENSDARG00000057859 | *hoxd10a* |
| 180448 | 1333 | 1,10731E-42 | ENSDARG00000057724 | *hoxa4a* |
| 51657 | 1333 | 2,05306E-24 | ENSDARG00000056819 | *hoxa9b* |
| 59326 | 1333 | 3,3279E-17 | ENSDARG00000010630 | *hoxb6a* |
| 55007 | 1333 | 8,39164E-16 | ENSDARG00000036254 | *hoxa13b* |
| 91786 | 1333 | 6,05517E-15 | ENSDARG00000031337 | *hoxa10b* |
| 125818 | 1333 | 7,14826E-14 | ENSDARG00000057724 | *hoxa4a* |
| 9589 | 1333 | 5,58475E-13 | ENSDARG00000031337 | *hoxa10b* |
| 32355 | 1333 | 3,01478E-11 | ENSDARG00000007609 | *hoxa13a* |
| 85022 | 1333 | 2,92553E-10 | ENSDARG00000070351 | *hoxc11a* |
| 103570 | 5887 | 3,87742E-71 | ENSDARG00000023031 | *hoxa2b* |
| 53135 | 5887 | 2,52762E-26 | ENSDARG00000059280 | *hoxd3a* |
