# Supplementary material for: Primitive Duplicate Hox Clusters in the European Eel's Genome
Source: PLoS One. 2012 Feb 24;7(2):e32231. doi: 10.1371/journal.pone.0032231 (PMC3286462; doi:10.1371/journal.pone.0032231)
Supplement: Table S3 — A. anguilla Hox genes. Complete list of A. anguilla Hox genes, predicted protein sizes, matching A. australis embryo contigs and best blastp hits. (DOC) [file pone.0032231.s006.doc]

**Table S3.** Complete list of *A. anguilla* *Hox* genes, predicted protein sizes, matching *A. australis* embryonic transcript contigs and best blastp hits.

| *A. anguilla* genome scaffold | *A. anguilla* *Hox* gene | Length of  ORF (#aa) | Transcriptome contig code(s) of 27h-old *A. australis* embryo with >98% identity to *A. anguilla Hox* gene; *bridges intron | Best blastp hit in *Danio rerio*-limited database (gene name; acc. number; E-value) | Best blastp hit in non-redundant database, excluding *Anguilla* sequences (gene name; species; accession number; E-value) |
| --- | --- | --- | --- | --- | --- |
| 5887 | *A1a* | 319 | 39608 | *A1a*; NP_571611.1; 6e-88 | *A1aa*; *Salmo salar*; NP_001133035.1; 1e-101 |
| 5887 | *A2a* | 366 | 103570* | *A2b*; NP_571181.1; 2e-146 | *A2*; *Latimeria menadoensis*; ACL81434.1; 3e-150 |
| 5887 | *A3a* | >330 | 53135* | *A3a*; Q8AWZ2.1; 1e-106 | *A3a*; *Oryzias latipes*; BAE44251.1; 2e-120 |
| 1333 | *A4a* | 241 | 28862, 180448 | *A4a*; NP_571610.1; 4e-93 | *A4a*; *Danio rerio*; NP_571610.1; 4e-91 |
| 1333 | *A5a* | 270 | 116346* | *A5a*; Q9YGT6.2; 6e-97 | *A5a*; *Haplochromis burtoni*; ABS70770.1; 1e-109 |
| 1333 | *A7a* | 236 | 3712, 96448, 152947 | *B7a*; NP_001108563.1; 3e-39 | *A7*; *Ornithorhynchus anatinus*; XP_001509365.1; 1e-66 |
| 1333 | *A9a* | 257 | 51657* | *A9a*; NP_571607.2; 6e-63 | *A9ab*; *Salmo salar*; NP_001133038.1; 2e-84 |
| 1333 | *A10a* | 308 | 9589, 91786, 159880 | *A10b*; NP_571230.1; 3e-95 | *A10*; *Latimeria menadoensis*; ACL81430.1; 4e-99 |
| 1333 | *A11a* | 269 | 43544, 85022 | *A11b*; NP_571222.1; 5e-108 | *A11ab*; *Salmo salar*; NP_001133037.1; 8e-107 |
| 1333 | *A13a* | >226 | 55007, 87149, 98005* | *A13a*; NP_001078963.1; 2e-65 | *A13*; *Latimeria menadoensis*; ACL81432.1; 1e-66 |
| 84 | *A1b* | 323 | 166269 | *A1a*; NP_571611.1; 8e-100 | *A1aa*; *Salmo salar*; NP_001133035.1; 1e-118 |
| 84 | *A2b* | 364 | 17128, 90864* | *A2b*; NP_571181.1; 9e-165 | *A2*; *Latimeria menadoensis*; ACL81434.1; 7e-172 |
| 84 | *A3b* | 412 | 55834*, 95324 | *A3a*; Q8AWZ2.1; 6e-97 | *A3a*; *Takifugu rubripes*; Q1KL12.1; 3e-109 |
| 84 | *A9b* | 258 | 52084* | *A9b*; Q9YGT5.3; 5e-79 | *A9*; *Heterodontus francisci*; Q9IA26.1; 2e-86 |
| 84 | *A10b* | 353 | 68514*, 106765 | *A10b*; NP_571230.1; 2e-116 | *A10*; *Callorhinchus milii*; ACU32546.1; 1e-118 |
| 84 | *A11b* | 281 | 15869, 34435, 130037, 138833*, 160656 | *A11b*; NP_571222.1; 8e-129 | *A11b*; *Devario aequipinnatus*; Q9DDU2.1; 8e-128 |
| 84 | *A13b* | 295 | none | *A13b*; P79724.3; 4e-143 | *A13; Latimeria menadoensis*; ACL81432.1; 5e-147 |
| 330 | *B1a* | 316 | 51405* | *B1a*; AAI62942.1; 3e-104 | *B1aa*; *Salmo salar*; NP_001133049.1; 4e-113 |
| 330 | *B2a* | 377 | 180588*, 128966 | *B2a*; NP_571191.1; 3e-145 | *B2aa*; *Salmo salar*; ABV82066.1; 2e-148 |
| 330 | *B3a* | 411 | 58468, 148360* | *B3a*; NP_571192.2; 7e-159 | *B3aa3*; *Salmo salar*; ABV82082.1; 1e-168 |
| 330 | *B4a* | 243 | 53043*, 80712, 86197, 162359 | *B4a*; NP_571193.1; 2e-110 | *B4a*; *Danio rerio*; NP_571193.1; 2e-108 |
| 330 | *B5a* | 279 | 46704*, 75377, 86079, 102356 | *B5a*; NP_571176.2; 1e-136 | *B5aa*; *Salmo salar*; ABW77467.1; 2e-136 |
| 330 | *B6a* | 227 | 104311* | *B6a*; CAD59113.1; 4e-116 | *B6a*; *Danio rerio*; CAD59113.1; 4e-114 |
| 330 | *B7a* | 220 | 145354* | *B7a*; NP_001108563.1; 8e-68 | *B7*; *Latimeria menadoensis*; ACL81450.1; 2e-75 |
| 330 | *B8a* | 248 | 103225* | *B8a*; NP_571195.1; 8e-127 | *B8ab*; *Salmo salar*; NP_001133051.1; 5e-127 |
| 330 | *B9a* | 249 | 179338* | *B9a*; Q9PWM2.2; 3e-122 | *B9ab*; *Salmo salar*; NP_001135098.1; 2e-128 |
| 330 | *B10a* | 286 | 51680* | *B10a*; AAI63689.1; 8e-57 | *B10aa*; *Salmo salar*; ABW77461.1; 1e-80 |
| 330 | *B13a* | 305 | none | *B13a*; NP_001038377.1; 3e-158 | *B13aa*; *Salmo salar*; NP_001133045.1; 3e-158 |
| 448 | *B1b* | 314 | 50994* | *B1a*; AAI62942.1; 2e-102 | *B1a*; *Megalobrama amblycephala*; ACN65052.1; 3e-107 |
| 472 | *B2b* | 374 | 50801* | *B2a*; NP_571191.1; 6e-147 | *B2aa*; *Salmo salar*; ABV82066.1; 8e-151 |
| 472 | *B3b* | 411 | 53472* | *B3a*; NP_571192.2; 1e-175 | *B3aa3*; *Salmo salar*; ABV82082.1; 3e-177 |
| 472 | *B4b* | 246 | 70145* | *B4a*; NP_571193.1; 4e-113 | *B4a*; *Megalobrama amblycephala*; ACN65055.1; 9e-112 |
| 472 | *B5b* | 275 | 81056*, 100250 | *B5a*; NP_571176.2; 2e-138 | *B5a*; *Danio rerio*; NP_571176.2; 3e-136 |
| 472 | *B6b* | 227 | 32927, 148750* | *B6a*; CAD59113.1; 1e-111 | *B6a*; *Danio rerio*; CAD59113.1; 1e-109 |
| 472 | *B7b* | 221 | 56431, 63358* | *B7a*; NP_001108563.1; 2e-82 | *B7*; *Latimeria menadoensis*; ACL81450.1; 7e-91 |
| 472 | *B8b* | 248 | 146468, 146756* | *B8a*; NP_571195.1; 1e-125 | *B8ab*; *Salmo salar*; NP_001133051.1; 7e-126 |
| 472 | *B9b* | 247 | 63828, 66055* | *B9a*; Q9PWM2.2; 4e-112 | *B9ab*; *Salmo salar*; NP_001135098.1; 3e-118 |
| 472 | *ψB10a* | pseudo | none | *C10a*; CAM16420.1; 3e-07 | *C10a*; *Haplochromis burtoni*; ABS70756.1; 4e-06 |
| 290 | *C1a* | 342 | none | *C1a*; NP_571606.1; 2e-35 | *C1*; *Latimeria menadoensis*; ACL81453.1; 3e-54 |
| 290 | *C4a* | 277 | 54638, 181614 | *C4a*; NP_571197.1; 1e-118 | *C4a*; *Danio rerio*; NP_571197.1; 1e-116 |
| 290 | *C5a* | 250 | 63328, 78426* | *C5a*; AAI63205.1; 9e-76 | *C5bb*; *Salmo salar*; NP_001133020.1; 3e-81 |
| 290 | *C6a* | 233 | 143448* | *C6a*; NP_571198.1; 2e-108 | *C6ba*; *Salmo salar*; NP_001135139.1; 1e-114 |
| 290 | *C8a* | 252 | 7133, 102927, 144813 | *C8a*; NP_001005771.1; 5e-120 | *C8ba*; *Salmo salar*; NP_001133013.1; 1e-125 |
| 290 | *C9a* | 261 | 55069* | *C9a*; AAD15960.1; 8e-116 | *C9aa*; *Salmo salar*; ABW77517.1; 3e-119 |
| 290 | *C10a* | 342 | 52095* | *C10a*; CAM16420.1; 1e-89 | *C10a*; *Danio rerio*; CAM16420.1; 2e-87 |
| 290 | *C11a* | 304 | none | *C11a*; NP_571240.1; 1e-145 | *C11a*; *Danio rerio*; NP_571240.1; 1e-143 |
| 290 | *C12a* | 275 | none | *C12a*; NP_001104229.1; 7e-132 | *C12a*; *Haplochromis burtoni*; ABS70754.1; 3e-133 |
| 290 | *C13a* | 307 | none | *C13a*; CAM14484.1; 9e-169 | *C13a*; *Danio rerio*; CAM14484.1; 1e-166 |
| 513 | *C4b* | 272 | 62505, 104558* | *C4a*; NP_571197.1; 6e-131 | *C4a*; *Danio rerio*; NP_571197.1; 7e-129 |
| 513 | *C5b* | 240 | 45462, 108663*, 188883 | *C5a*; AAI63205.1; 7e-75 | *C5a*; *Danio rerio*; AAI63205.1; 8e-73 |
| 513 | *C6b* | 233 | 103504* | *C6a*; NP_571198.1; 6e-108 | *C6a*; *Oryzias latipes*; BAE44280.1; 2e-109 |
| 513 | *C8b* | 246 | 143804 | *C8a*; NP_001005771.1; 3e-122 | *C8ba*; *Salmo salar*; NP_001133013.1; 4e-125 |
| 513 | *C9b* | 261 | 144798*, 179450 | *C9a*; AAD15960.1; 2e-121 | *C9a*; *Haplochromis burtoni*; ABS70757.1; 5e-123 |
| 513 | *C10b* | 339 | 93391, 100662* | *C10a*; CAM16420.1; 3e-107 | *C10a*; *Danio rerio*; CAM16420.1; 4e-105 |
| 513 | *C11b* | 307 | none | *C11a*; NP_571240.1; 4e-156 | *C11a*; *Danio rerio*; NP_571240.1; 5e-154 |
| 513 | *C12b* | 274 | none | *C12a*; NP_001104229.1; 2e-132 | *C12a*; *Danio rerio*; NP_001104229.1; 3e-130 |
| 513 | *C13b* | 307 | none | *C13a*; CAM14484.1; 1e-173 | *C13a*; *Danio rerio*; CAM14484.1; 1e-171 |
| 905 | *D1a* | 296 | 143376* | *A1a*; AAD15937.1; 2e-35 | *D1aa*; *Salmo salar*; NP_001133027.1; 7e-66 |
| 905 | *D3a* | 396 | 103979, 180487 | *D3a*; NP_571200.1; 0.0 | *D3a*; *Danio rerio*; NP_571200.1; 0.0 |
| 905 | *D4a* | 236 | 56200* | *D4a*; NP_001119917.1; 8e-118 | *D4aa*; *Salmo salar*; ABV82026.1; 1e-118 |
| 905 | *D8a* | 234 | 24507, 84846* | *C8a*; NP_001005771.1; 2e-82 | *D8*; *Scyliorhinus canicula*; CBL59362.1; 8e-102 |
| 905 | *D9a* | 263 | 51991* | *D9a*; NP_571201.3; 5e-122 | *D9aa*; *Salmo salar*; ABW77554.1; 9e-125 |
| 905 | *D10a* | 334 | 33331, 55695*, 125941 | *D10a*; NP_571241.2; 9e-154 | *D10aa*; *Salmo salar*; NP_001133025.1; 5e-170 |
| 905 | *D11a* | 265 | none | *D11a*; AAI14272.1; 7e-124 | *D11aa*; *Salmo salar*; NP_001133024.1; 2e-123 |
| 905 | *D12a* | 258 | none | *D12a*; Q90471.2; 2e-89 | *D12aa*; *Salmo salar*; NP_001133023.1; 3e-101 |
| 905 | *D13a* | 289 | none | *D13a*; AAI53653.1; 8e-62 | *D13aa*; *Salmo salar*; NP_001133022.1; 2e-76 |
| 1005 | *ψD3b* | pseudo | 87144 | *D3a*; NP_571200.1; 0.005 | *D3*; *Latimeria menadoensis*; ACL81468.1; 0.008 |
| 1005 | *D4b* | 231 | 56644, 57327 | *D4a*; NP_001119917.1; 8e-105 | *D4a*; *Danio rerio*; NP_001119917.1; 9e-103 |
| 1005 | *ψD8b* | pseudo | none | *C8a*; NP_001005771.1; 0.35 | *D8*; *Heterodontus francisci*; Q9IA12.1; 0.008 |
| 1005 | *D9b* | 263 | 53985* | *D9a*; NP_571201.3; 1e-114 | *D9a*; *Osmerus mordax*; ACO09462.1; 1e-115 |
| 1005 | *D10b* | 333 | 83317, 189155 | *D10a*; NP_571241.2; 5e-141 | *D10aa*; *Salmo salar*; NP_001133025.1; 5e-151 |
| 1005 | *D11b* | 279 | none | *D11a*; AAI14272.1; 7e-124 | *D11a*; *Danio rerio*; AAI14272.1; 9e-122 |
| 1005 | *D12b* | 254 | none | *D12a*; Q90471.2; 4e-80 | *D12aa*; *Salmo salar*; ABW77551.1; 1e-86 |
