# Supplementary material for: Primitive Duplicate Hox Clusters in the European Eel's Genome
Source: PLoS One. 2012 Feb 24;7(2):e32231. doi: 10.1371/journal.pone.0032231 (PMC3286462; doi:10.1371/journal.pone.0032231)
Supplement: Table S4 — Hox genes used in phylogeny reconstruction. List of the Hox gene sequences used in this study. (DOC) [file pone.0032231.s007.doc]

**Table S4.** List of the Hox homologues used in this study.

**Hox9 genes (Figure 3)**

————————————————————————————————————————

Species (common name) Gene Accession no.

————————————————————————————————————————

*Latimeria menadoensis* (Indonesian coelacanth) *HoxA9* ACL81440

*HoxB9* ACL81452

*HoxC9* ACL81463

*HoxD9* ACL81471

*Danio rerio* (zebrafish) *HoxA9a* NP_571607

*HoxA9b* NP_571608

*HoxB9a* NP_571196

*HoxC9a* NP_571603

*HoxD9a* NP_571201

*Megalobrama amblycephala* (Wuchang bream) *HoxB9a* ABM73566

*Esox lucius* (northern pike) *HoxD9a* ACO13802

*Salmo salar* (Atlantic salmon) *HoxA9aa* ABW77440

*HoxA9ab* NP_001133038

*HoxA9b* NP_001133044

*HoxB9aa* ABW77463

*HoxB9ab* NP_001135098

*HoxC9aa* NP_001132998

*HoxC9ab* NP_001133005

*HoxC9ba* NP_001133012

*HoxC9bb* NP_001133018

*HoxD9aa* NP_001135141

*HoxD9ab* NP_001135095

*Osmerus mordax* (rainbow smelt) *HoxD9a* ACO09462

*Oryzias latipes* (Japanese medaka) *HoxA9a* BAE44255

*HoxA9b* BAE44260

*HoxB9a* BAE44271

*HoxC9a* BAE44282

*HoxD9a* BAE53499

*HoxD9b* BAE53502

*Gasterosteus aculeatus* (stickleback) *HoxD9a* AY744145.1

*Morone saxatilis* (striped sea-bass) *HoxA9* AAD46396

*Haplochromis burtoni* *HoxA9a* ABS70772

*HoxA9b* ABS70743

*HoxB9a* ABS70738

*HoxC9a* ABS70757

*HoxD9a* ABS70794

*HoxD9b* ABS70798

*Takifugu rubripes* (takifugu) *HoxA9a* ABF22379

*HoxA9b* ABF22401

*HoxB9a* ABF22412

*HoxC9a* ABF22444

*HoxD9a* ABF22465

*HoxD9b* ABF22477

————————————————————————————————————————

**Hox9 genes (Figure S2)**

————————————————————————————————————————

Species (common name) Gene Accession no.

————————————————————————————————————————

*Latimeria menadoensis* (Indonesian coelacanth) *HoxA9* ACL81440

*HoxB9* ACL81452

*HoxC9* ACL81463

*HoxD9* ACL81471

*Xenopus tropicalis* (Western clawed frog) *HoxA9* ENSXETP00000001611

*HoxB9* ENSXETP00000025572

*HoxC9* ENSXETP00000062221

*HoxD9* ENSXETP00000016495

*Homo sapiens* (human) *HoxA9* NP_689952

*HoxB9* NP_076922

*HoxC9* NP_008828

*HoxD9* NP_055028

*Danio rerio* (zebrafish) *HoxA9a* NP_571607

*HoxA9b* NP_571608

*HoxB9a* NP_571196

*HoxC9a* NP_571603

*HoxD9a* NP_571201

*Salmo salar* (Atlantic salmon) *HoxA9aa* ABW77440

*HoxA9ab* NP_001133038

*HoxA9b* NP_001133044

*HoxB9aa* ABW77463

*HoxB9ab* NP_001135098

*HoxC9aa* NP_001132998

*HoxC9ab* NP_001133005

*HoxC9ba* NP_001133012

*HoxC9bb* NP_001133018

*HoxD9aa* NP_001135141

*HoxD9ab* NP_001135095

*Oryzias latipes* (Japanese medaka) *HoxA9a* NP_001098136

*HoxA9b* NP_001098135

*HoxB9a* BAE44271

*HoxC9a* NP_001188421

*HoxD9a* BAE44289

*HoxD9b* BAE44294

*Tetraodon nigroviridis* (green spotted puffer) *HoxA9a* ENSTNIP00000000288

*HoxA9b* ENSTNIP00000004131

*HoxB9a* ENSTNIP00000013750

*HoxC9a* ENSTNIP00000004124

*HoxD9a* ENSTNIP00000020079

*HoxD9b* ENSTNIP00000004143

**Hox genes (Figure 4)**

————————————————————————————————————————

Species (common name) Gene Accession no.

————————————————————————————————————————

*Danio rerio* (zebrafish) *HoxA9a* NP_571607

*HoxA9b* NP_571608

*HoxA11a* NP_571619

*HoxA11b* NP_571222

*HoxA13a* NP_001078963

*HoxA13b* NP_571269

*HoxB1a* NP_571190

*HoxB1b* NP_571217

*HoxB5a* NP_571176

*HoxB5b* NP_571612

*HoxB6a* NP_571194

*HoxB6b* NP_571613

*HoxC6a* NP_571198

*HoxC6b* NP_571605

*HoxC11a* NP_571240

*HoxC11b* XP_699549

*HoxC12a* NP_001104229

*HoxC12b* NP_571620

*HoxC13a* NP_571618

*HoxC13b* NP_571621

*HoxD4a* NP_001119917

*HoxD9a* NP_571201

*Salmo salar* (Atlantic salmon) *HoxA9aa* ABW77440

*HoxA9ab* NP_001133038

*HoxA9b* NP_001133044

*HoxA11aa* NP_001133032

*HoxA11ab* NP_001133037

*Hox-A11b* NP_001135144

*HoxA13aa* NP_001135148

*HoxA13ab* NP_001133036

*Hox-A13b* NP_001133042

*HoxB1aa*  NP_001133049

*HoxB1ab*  NP_001133055

*HoxB1ba* ABW77490

*HoxB3aa* NP_001133048

*HoxB5aa* NP_001135145

*HoxB5ba* ABW77488

*HoxB5bb* ABW77507

*HoxB6aa* NP_001133047

*HoxB6ab* NP_001133052

*HoxB6ba*  NP_001135147

*HoxB6bb*  ABW77506

*HoxC6aa* NP_001133000

*HoxC6ab* NP_001133007

*HoxC6ba*  NP_001135139

*HoxC6bb*  NP_001135094

*HoxC11aa*  NP_001132997

*HoxC11ab* NP_001135137

*HoxC11ba* ABW77533

*HoxC11bb*  NP_001133017

*HoxC12aa* ABW77514

*HoxC12ab* NP_001135091

*HoxC12ba* NP_001133010

*HoxC12bb* NP_001133016

*HoxC13aa*  NP_001132996

*HoxC13ab*  NP_001133003

*HoxC13ba*  NP_001135138

*HoxC13bb*  NP_001133015

*HoxD4aa*  ABW77555

*HoxD4ab*  NP_001133031

*HoxD9aa* NP_001135141

*HoxD9ab* NP_001135095

*Oryzias latipes* (Japanese medaka) *HoxA9a* NP_001098136

*HoxA9b* NP_001098135

*HoxA11a* BAE44257

*HoxA11b* BAE44262

*HoxA13a* BAE44258

*HoxA13b* BAE44263

*HoxB1a* BAE44264

*HoxB1b* BAE44273

*HoxB5a* BAE44268

*HoxB5b* BAE44275

*HoxB6a* BAE44269

*HoxB6b*  BAE44276

*HoxC6a*  BAE44280

*HoxC11a*  BAE44284

*HoxC12a* BAE44285

*HoxC13a* BAE44286

*HoxD4a* BAE44288

*HoxD4b*  BAE44293

*HoxD9a* BAE44289

*HoxD9b* BAE44294

*Tetraodon nigroviridis* (green spotted puffer) *HoxA9a* ENSTNIP00000000288

*HoxA9b* ENSTNIP00000004131

*HoxA11a* ENSTNIP00000004129

*HoxA11b* ENSTNIP00000003617

*HoxA13a* ENSTNIP00000012067

*HoxA13b* ENSTNIP00000004145

*HoxB1a* ENSTNIP00000005927

*HoxB1b* ENSTNIP00000012991

*HoxB5a* ENSTNIP00000004117

*HoxB5b* ENSTNIP00000004133

*HoxB6a* ENSTNIP00000012989

*HoxB6b* ENSTNIP00000004134

*HoxC6a* ENSTNIP00000004122

*HoxC11a* ENSTNIP00000004136

*HoxC12a* ENSTNIP00000004137

*HoxC13a* ENSTNIP00000004138

*HoxD4a* ENSTNIP00000004126

*HoxD4b* ENSTNIP00000004142

*HoxD9a* ENSTNIP00000020079

*HoxD9b* ENSTNIP00000004143
