# Supplementary material for: Primitive Duplicate Hox Clusters in the European Eel's Genome
Source: PLoS One. 2012 Feb 24;7(2):e32231. doi: 10.1371/journal.pone.0032231 (PMC3286462; doi:10.1371/journal.pone.0032231)
Supplement: Table S5 — Hox clusters used in synteny analysis. Genomic locations of D. rerio and O. latipes Hox clusters. HoxCb is absent from O. latipes, and HoxDb from D. rerio. However, the genomic loci can still be identified based on the presence of flanking gene duplicates or conserved microRNA (D. rerio HoxDb). (DOC) [file pone.0032231.s008.doc]

**Table S5.** Hox loci in zebrafish and medaka.

| Species | Assembly | Cluster | Chromosome | Location | Notes |
| --- | --- | --- | --- | --- | --- |
| *D. rerio* | Zv9 | Aa | 19 | 19195251–19253497 |  |
| *D. rerio* | Zv9 | Ab | 16 | 22980334–23013613 |  |
| *D. rerio* | Zv9 | Ba | 3 | 23945474–24062383 |  |
| *D. rerio* | Zv9 | Bb | 12 | 28688847–28713862 |  |
| *D. rerio* | Zv9 | Ca | 23 | 36104488–36235041 |  |
| *D. rerio* | Zv9 | Cb | 11 | 2108753–2180948 | 1 |
| *D. rerio* | Zv9 | Da | 9 | 1927848–1981122 |  |
| *D. rerio* | Zv9 | Db | 6 | 10777971–10778088 | 2 |
| *O. latipes* | MEDAKA1 | Aa | 11 | 10493938–10563229 |  |
| *O. latipes* | MEDAKA1 | Ab | 16 | 13115192–13137446 |  |
| *O. latipes* | MEDAKA1 | Ba | 8 | 24280190–24441968 |  |
| *O. latipes* | MEDAKA1 | Bb | 19 | 17579481–17594165 |  |
| *O. latipes* | MEDAKA1 | Ca | 7 | 12836622–12915920 |  |
| *O. latipes* | MEDAKA1 | Cb | 5 | 27900000 | 3 |
| *O. latipes* | MEDAKA1 | Da | 21 | 24601819–24637177 |  |
| *O. latipes* | MEDAKA1 | Db | 15 | 4352002–4374365 |  |

1 Possibly misassembled: HoxC13b anterior to HoxC6b, as well as in the opposite orientation; flanking genes spanning or invading the Hox cluster.

2 Putative locus, no Hox genes (based on miR-10d).

3 Putative locus, no Hox genes.
